# Insights into the molecular basis of the NOD2 signalling pathway

**DOI:** 10.1098/rsob.140178

**Published:** 2014-12-17

**Authors:** Joseph P. Boyle, Rhiannon Parkhouse, Tom P. Monie

**Affiliations:** 1 Department of Biochemistry, University of Cambridge, Cambridge, UK; 2 Department of Veterinary Medicine, University of Cambridge, Cambridge, UK; 3 MRC Human Nutrition Research, Elsie Widdowson Laboratory, 120 Fulbourn Road, Cambridge, UK

**Keywords:** NLR, innate immunity, signal transduction, post-translational modification, RIP2 kinase, NOD1/2

## Abstract

The cytosolic pattern recognition receptor NOD2 is activated by the peptidoglycan fragment muramyl dipeptide to generate a proinflammatory immune response. Downstream effects include the secretion of cytokines such as interleukin 8, the upregulation of pro-interleukin 1β, the induction of autophagy, the production of antimicrobial peptides and defensins, and contributions to the maintenance of the composition of the intestinal microbiota. Polymorphisms in NOD2 are the cause of the inflammatory disorder Blau syndrome and act as susceptibility factors for the inflammatory bowel condition Crohn's disease. The complexity of NOD2 signalling is highlighted by the observation that over 30 cellular proteins interact with NOD2 directly and influence or regulate its functional activity. Previously, the majority of reviews on NOD2 function have focused upon the role of NOD2 in inflammatory disease or in its interaction with and response to microbes. However, the functionality of NOD2 is underpinned by its biochemical interactions. Consequently, in this review, we have taken the opportunity to address the more ‘basic’ elements of NOD2 signalling. In particular, we have focused upon the core interactions of NOD2 with protein factors that influence and modulate the signal transduction pathways involved in NOD2 signalling. Further, where information exists, such as in relation to the role of RIP2, we have drawn comparison with the closely related, but functionally discrete, pattern recognition receptor NOD1. Overall, we provide a comprehensive resource targeted at understanding the complexities of NOD2 signalling.

## 2. Introduction

**Figure 1.**
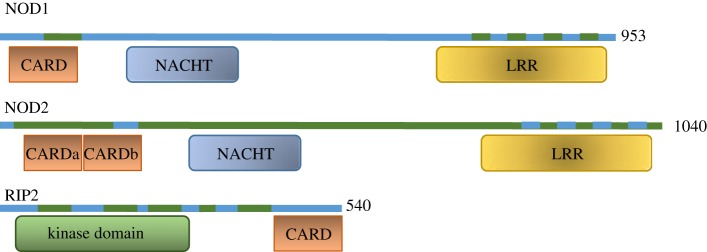
The domain architecture of NOD1, NOD2 and RIP2. Exons are shown in alternating blue and green blocks. Protein domains as listed in the NCBI RefSeq database are shown in boxes (Accession numbers: NOD1—NP_006083; NOD2—NP_07115; RIP2—NP_003812). The length of each protein is shown. CARD, caspase recruitment domain; NACHT, NAIP, CIITA, HET-E and TP1-containing; LRR, leucine-rich repeats.

Nucleotide-binding and oligomerization-domain containing 2 (NOD2) was the second member of the nucleotide-binding domain and leucine-rich repeat containing receptor (NLR) family to be identified [1], following the discovery of NOD1 in 1999 [2]. These two receptors have similar domain architectures—a C-terminal leucine-rich repeat (LRR) domain, a central NAIP, CIITA, HET-E and TP1-containing (NACHT) domain and an N-terminal effector domain. The effector region consists of one caspase recruitment domain (CARD) in NOD1 and two tandem CARDs in NOD2 (figure 1). Based on the recent structure of NLRC4 [3] (PDB ID: 4KXF), it can be predicted that the NACHT domains in NOD1 and NOD2 are followed by a proximal helical domain (HD1), a winged-helix domain (WH) and a distal helical domain (HD2).

These structural similarities accompany similarity in function—NOD1 and NOD2 are believed to be held in an autoinhibited state by their LRRs, are activated by peptidoglycan fragments, bind nucleotides and oligomerize through their NACHT domains and engage the downstream signalling molecule receptor-interacting protein 2 (RIP2) through their effector domains [4].

**Figure 2.**
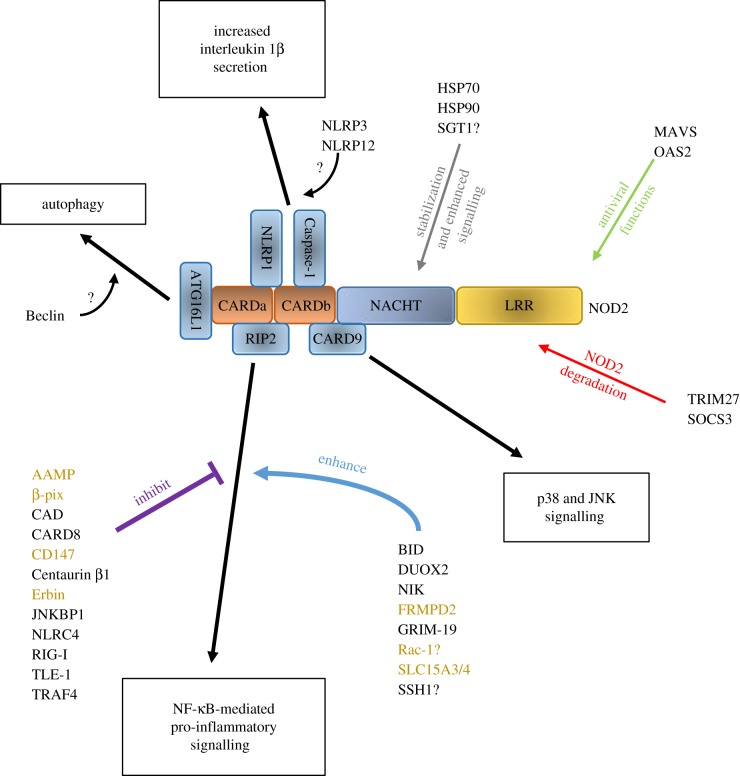
NOD2-interaction partners influence a wide range of NOD2 functions. A schematic of the reported interactions between NOD2 and other cellular proteins. For the sake of simplicity, only a selection of proteins are displayed in direct contact with NOD2. Key NOD2 functional outputs are shown in black boxes. Protein partners influencing these functions directly are listed in the relevant location. Protein impacts on NOD2 are highlighted with coloured arrows. Proteins which exert their influence and interact with NOD2 at a predominantly membrane location are labelled in gold. Where the precise role of a protein partner is uncertain this is represented by a question mark, and the protein has been located in the most likely region of influence.

Despite these similarities, research on NOD2 has been more prominent, predominantly owing to the identification of numerous NOD2 single nucleotide polymorphisms (SNPs) which are associated with Crohn's disease or causal for Blau syndrome. The role of NOD2 in these diseases has been illuminated by the identification of proteins, such as ATG16L1 and CARD9, which are both linked to Crohn's disease [5,6] and interact with NOD2 [7–9]. As well as these binding partners NOD2 has been reported to bind a wide variety of other proteins (table 1 and figure 2). The identification of similarities between subgroups of these proteins may provide insights into the physiological roles of NOD2 and its contributions to disease. In this review, we provide a comprehensive reference table of currently reported NOD2 binding partners and discuss the role and contribution of these to NOD2 signalling (table 1).

**Table 1.** NOD2 binding partners. Where the effects of NOD1 and NOD2 differ, the row is split.

protein name	binds	interaction localization	binding domain	effect of interaction	linked to disease?
AAMP	binds NOD1 weakly [10]			inhibits NF-κB signalling [10]	
	binds NOD2 [10]	cytoplasm, plasma membrane [10]	NACHT, CARDs [10]	inhibits NF-κB signalling [10]	
ATG16L1	binds NOD1 [7,11]	plasma membrane, bacterial entry sites [7]	CARD [11]	promotes autophagy [7]	Crohn's disesase [5]
	BINDS NOD2 [7,11,12]	plasma membrane, bacterial entry sites [7]	CARDa [12]; Tandem CARDs [11]	promotes autophagy [7]	
β-PIX	binds NOD2 [13]	plasma membrane [13]		membrane recruitment, inhibits IL-8 production [13]	
Beclin	binds NOD2 [14]				
BID	binds NOD1 [15]; binds NOD2 [15]			enhances NF-κB and ERK signalling [15]	
CAD	binds NOD1 [16]			inhibits NF-κB signalling [16]	
	binds NOD2 [16]		CARDs [16]	inhibits NF-κB signalling [16]	
CARD8	binds NOD2 [17]	cytoplasm [17]	NACHT [17]	inhibits NF-κB signalling [17]	Crohn's disease [18]
CARD9	binds NOD2 [8]		CARD–NACHT linker, NACHT [9]	enhances p38 signalling [8]	Crohn's disease [6]
Caspase-1	binds NOD1 [19]		CARD [19]	enhances IL-1β secretion [19]	
	binds NOD2 [20]		Tandem CARDs [20]	enhances IL-1β secretion [20]	
CD147	binds NOD2 [21]	plasma membrane [21]	Tandem CARDs [21]	inhibits NF-κB and IL-8 signalling [21]	
Centaurin-β1	binds NOD1 [22]; binds NOD2 [22]	cytoplasm [22]		inhibits NF-κB [22]	
DUOX2	binds NOD2 [23]	cytoplasm, membrane [23]	LRR [23]	enhances NF-κB [23]	
Erbin	does not bind NOD1 [24,25]				
	binds NOD2 [24,25]	plasma membrane [24,25]	CARDs [24]	inhibits NF-κB [24]	
FRMPD2	binds NOD2 [26]	plasma membrane [26]	LRR [26]	membrane recruitment, enhances NF-κB [26]	
GRIM-19	binds NOD2 [27]	intracellular vesicles [27]		enhances NF-κB [27]	
	does not bind NOD1 [27]				
HSP70	binds NOD2 [28]			promotes NOD2 stability; enhances NF-κB signalling [28]	
HSP90	binds NOD1 [29,30]			promotes NOD1 stability [29]	
	binds NOD2 [30,31]		CARDs [31]	stabilizes NOD2, required for NF-κB signalling [31]	
JNKBP1	does not bind NOD1 [32]				
	binds NOD2 [32]		CARDa, LRRs [32]	inhibits NF-κB and IL-8 signalling [32]	
MAVS	does not bind NOD1 [33]				
	binds NOD2 [33]	mitochondria [33]	NACHT-LRR [33]	antiviral defence [33]	
NIK	binds NOD2 [34]		NACHT-LRR [34]	enhances non-canonical NF-κB signalling [34]	
NLRC4	binds NOD1 [35]		NACHT [35]	inhibits NF-κB [35]	
	binds NOD2 [36,35]		CARD [36], NACHT [35]	inhibits NF-κB [35]	
NLRP1	binds NOD2 [20,37]		CARDs [37]	enhances IL-1β secretion [20]	Crohn's disease [38]
NLRP3	binds NOD2 [37]		CARDs [37]		Crohn's disease [38]
NLRP12	binds NOD2 [37]		CARDs [37]		
OAS2	binds NOD2 [39]			enhances RNase-L function [39]	
Rac1	binds NOD2 [13,40]	plasma membrane [13,40]	CARDS, LRR [40]	membrane recruitment [13,40]	
RIG-I	binds NOD2 [41]	membrane ruffles [41]		inhibits NF-κB signalling [41]	
RIP2	binds NOD1 [19]		CARD [19]	required for NF-κB signalling [42]	
	binds NOD2 [1]	plasma membrane [43]	CARDs [1]	required for NF-κB signalling [42]	
SGT1	binds NOD1 [44,30]		LRR [44]	enhances NF-κB and JNK signalling [44]	
	binds NOD2 [44,30]			no effect on NF-κB or JNK signalling [44]	
SLC15A3/4	binds NOD2 [45]	endosomal membrane [45]		enhances NF-κB signalling [45]	
SOCS-3	binds NOD2 [31]		CARDs [31]	NOD2 degradation [31]	
SSH1	binds both NOD1 and NOD2 [46]	actin complexes		regulation of NOD1 signalling [46]	
TLE1	binds NOD2 [47]	perinuclear cytosol [47]		inhibits NF-κB signalling [47]	Crohn's disease [47]
TRAF4	binds NOD1 [48]				
	binds NOD2 [48]		residues 260–301 [48]	inhibits NF-κB signalling [48]	
TRIM27	Does not bind NOD1 [49]				
	Binds NOD2 [49]	nucleus [49]	NACHT [49]	NOD2 degradation [49]

## 3. The stability, autoinhibition and degradation of NOD2

NOD2 is maintained in an inactive, autoinhibited conformation in the cell through interactions between the NACHT and LRR domains and interaction with cellular chaperones. Hahn [29] originally proposed that NOD1 interacts with the chaperone protein heat shock protein 90 (HSP90), which provides stability in a manner analogous to some plant R proteins. This hypothesis was extended to NOD2 by da Silva Correia *et al.* [44], who used HSP90 siRNA and a small molecule HSP90 inhibitor, geldanamycin, to show the importance of this chaperone for NOD1 and NOD2 stability in MCF-7 cells. This study also went on to investigate the role of SGT1, another regulator of R protein activity. While SGT1 bound NOD1 and NOD2, it was only important for cytokine signalling through NOD1 and its knockdown by siRNA did not affect the stability of either protein. Not long after, a study by Mayor *et al.* [30] implicated SGT1 and HSP90 as important for NOD2 signalling, though experimentation was restricted to the inhibitory effects of geldanamycin on muramyl dipeptide (MDP) signalling.

The association between HSP90 and NOD2 was reproduced by Lee *et al.* [31], who confirmed its importance for NOD2 stability. In this case, HSP90 was suggested to act as part of a negative feedback loop, wherein activation of NOD2 causes its dissociation from HSP90 and subsequent, proteasome-dependent degradation. The role of HSP90 was again confirmed using small molecule inhibitors, while the proteasome inhibitor MG-132 blocked NOD2 degradation [31]. As well as dissociation from HSP90, the negative feedback loop was shown to involve SOCS3, a NOD2-binding, MDP-inducible protein which was at least partially responsible for the ubiquitination of NOD2 leading to proteasomal degradation [31]. SOCS3 and HSP90 both bound to the CARDs of NOD2 and so may be mutually exclusive complexes, and it was hypothesized that SOCS3 could link NOD2 to an as yet unknown E3 ligase. A candidate E3 ligase for this process is TRIM27, which has been reported to be important for the ubiquitination and degradation of NOD2 [49].

More recently, HSP70 has also been shown to interact with, and stabilize, NOD2 [28]. In this work, overexpression of HSP70 resulted in an increase in NF-κB activity following ligand-mediated stimulation of NOD2. In contrast, reducing HSP70 levels through the use of the small molecule inhibitor KNK347 led to associated reduction in NF-κB signalling. Analysis of NOD2 protein levels demonstrated that HSP70 was acting to stabilize the NOD2 protein, as when HSP70 levels were reduced the half-life of NOD2 decreased [28]. It remains to be seen whether all these chaperones are acting in concert with one another or whether they can promote NOD2 stability independently.

## 4. Recognition of ligand by NOD2

NOD2 is the bona fide cytoplasmic receptor for the peptidoglycan fragment MDP [50]. The introduction of MDP into the cytoplasm can be achieved by multiple pathways including: peptide transporters SLC15A1, 3 and 4 [45,51–53]; invasive bacteria, such as *Shigella flexneri,* shedding peptidoglycan [54]; and the absorption of outer membrane vesicles released from Gram-negative bacteria [55,56].

Using both biophysical and biochemical approaches, it has been shown recently that MDP interacts directly with NOD2 [57,58]. It is generally believed that recognition is mediated by the NOD2 LRRs [4,57,59,60] although a critical role for the NACHT region has been suggested [58], though this may represent a requirement for correct LRR folding in the cell.

NOD2 can also undergo autoactivation, and this is observed in the rare, autosomal dominant, inflammatory disorder Blau syndrome which is caused by NOD2 polymorphisms [61]. NOD2 SNPs that cause Blau syndrome cluster into two regions of NOD2—the nucleotide/Mg^2+^ binding pocket, and helical domain 1 between the NACHT and LRR. This has led to the suggestion that the mechanism by which autoactivation occurs is likely to result from either interference with nucleotide binding and hydrolysis, or interference with chaperone binding and the intramolecular contacts between the NOD2 NACHT and helical domain 1 with the LRR [62].

## 5. NOD2 and the adaptor protein RIP2

RIP2 is the best-studied interaction partner of NOD2 and is important for the activation of the NF-κB [42,63] and MAPK pathways [64,65] by NOD2 and also NOD1 [42]. Although the importance of RIP2 in NOD signalling has been well demonstrated, many questions remain open regarding its interaction with NOD1 and NOD2, its precise role in MAPK signalling and autophagy, the purpose of its kinase domain and the role of its many post-translational modifications.

The domain architecture of RIP2 comprises an N-terminal kinase domain, a central linker region and a C-terminal CARD (figure 1). Structural information is currently available for its kinase domain (PDB ID: 4C8B), which like RIP1 and RIP3 shows a typical kinase fold. The C-terminal CARD of RIP2 is expected to form a six-helix bundle in accordance with the rest of the death domain superfamily, whereas the central region is thought to be broadly unstructured and highly flexible.

Overexpression of the tandem NOD2 CARDs is sufficient to give a constitutive NF-κB response, but this does not happen with either NOD2 CARD individually [1]. In line with this, neither NOD2 CARD alone is able to bind RIP2 [1,37] and specific point mutation within either of the NOD2 CARDs abrogates RIP2 binding and prevents NF-κB signalling [4]. Accordingly, the shortest section of NOD2 reported to bind to RIP2 is NOD2-S [66], which contains CARDa and the first three helices of CARDb. Interestingly, despite being able to bind RIP2, overexpression of NOD2-S does not activate NF-κB. Taken together, these observations suggest that the engagement of RIP2 by NODs is not sufficient for NF-κB activation and would be consistent with recent observations of NOD1 and RIP2 [67]. Indeed, complete activation of RIP2-mediated NOD2 signalling may require: the complex to assemble with a specific conformation; post-translational modification; simultaneous interaction between the NOD2 CARDs themselves or the binding of another essential protein. While the NOD1 and NOD2 CARDs have both been shown to self-associate [37,67–69], it is not yet clear whether these interactions are physiologically relevant. In addition, as discussed in this review, various other proteins have been shown to interact with NOD2 and to be crucial for successful activation of NF-κB.

The residues which mediate NOD1, NOD2 and RIP2 complex formation are controversial and are complicated firstly by the possibility that multiple interfaces may be involved in CARD : CARD interactions and secondly by the potential structural roles of charged, surface residues in CARDs. A model for the CARD : CARD interaction between NOD1 and RIP2 was proposed by Manon *et al.* [70] in 2007 following their solution of the NOD1 CARD structure by NMR (PDB ID: 2B1W). This structure, combined with a homology model of RIP2 based on the caspase-9 CARD, proposed two opposing charged surfaces, one acidic on NOD1 and one basic on RIP2, which interact to form a heterodimer. However, this original NMR model differs significantly from subsequent crystallographic and NMR NOD1 CARD structures [69,71,72].

Site-directed mutagenesis resulted in a model postulating that three core acidic residues—E53, D54 and E56—formed the core acidic patch on NOD1, whereas R444, R483 and R488 formed the RIP2 basic patch. Charge reversal mutants of any one of these residues were able to disrupt the binding of NOD1 to RIP2 almost completely, although the mutation E56A has no impact on NOD1 signalling [60]. A charge reversal mutation of R69 in NOD1 was also able to disrupt the NOD1 : RIP2 interaction, though this was suggested to be due to a destabilization of the CARD fold [70]. More recently, it has been shown that the interaction between NOD1 and RIP2 may involve both a type I interface consisting of residues centred around R38, R69 and R86 on NOD1 and D461 and Y474 on RIP2; as well as a type III interface centred upon E53 and E56 on NOD1 and R483 on RIP2 [67]. This study also demonstrated that the importance of D54 in NOD1 signalling and the engagement of RIP2 resulted from its key role in stabilizing the NOD1 CARD structure through formation of an intramolecular salt bridge with K78.

The core acidic trio identified in NOD1 is conserved in NOD2 (E69, D70 and E72). However, there is contrasting evidence regarding their role in RIP2 binding. Wagner *et al.* [37] showed that E69K and D70K mutants disrupt RIP2 binding in a yeast two-hybrid study, whereas Fridh *et al.* [68] generated the E69K and E72K mutants in *E. coli* constructs and found that they are not necessary for RIP2 binding, instead proposing that a basic patch on NOD2 CARDa interacts with an acidic patch on the RIP2 CARD. These differences may result from the utilization of different CARD : CARD interfaces and the preference of different CARD : CARD complexes under certain experimental conditions. However, it is possible that the loss of interaction results from introduction of a secondary mutation that destabilizes the protein fold, rather than disrupting an interaction surface. Certainly, different groups have reported varying levels of difficulty in expressing certain NOD1 and NOD2 mutants in *E. coli*, suggesting that certain charged residues may contribute to the stability of the CARD fold under specific conditions. For example, Manon *et al.* [70] reported that the NOD1 R69E mutant is unstable; Fridh *et al.* [68] showed that the NOD2 D58A, D70A, L145P, E166K and R182A mutants gave especially low expression; and Ver Heul *et al.* [11] reported that E53K, D54K and E56K mutants could not be expressed in *E. coli*, although Mayle *et al.* [67] successfully produced both E53K and E56K.

These reports indicate that the mutation of numerous residues in the CARDs may be important for correct folding and that such residues may exist outside the hydrophobic core. Studies using mammalian cells may be especially prone to misinterpreting the roles of surface residues by not taking into account slight or severe disruptions in the protein fold. For example, early work on NOL3 and Bcl-10 CARD function mutated different residues within their hydrophobic cores (L31F and L41Q, respectively) to use as negative controls [73,74]. These proteins still gave normal expression in HEK293 cells but had lost function, indicating that even severe, internal mutations can be tolerated without preventing protein overexpression. As such, the structural importance of surface residues should be strongly considered in mutational studies of CARDs. Future mutational work and structural evidence will be required to clarify the stoichiometry and mode of interaction between NOD2 and RIP2.

## 6. The importance of post-translational modification of RIP2 in NOD2 signalling

Functional analysis of RIP2 and broad studies of the protein kinome have revealed that RIP2 is highly phosphorylated (table 2). Phosphorylation of RIP2 tends to occur on exposed, flexible regions. The two phosphoserine residues in the RIP2 kinase domain, S168 and S176, fall into an unsolved region in the RIP2 crystal structure and the homologous residues in the RIP1 and RIP3 crystal structures are also absent. Two phosphoserines detected by large-scale kinome analysis, S363 and S393, are found in the interdomain region of RIP2 alongside Y381. Meanwhile, Y520, which borders the predicted end of the CARD, is between two proline residues, and the flexible C-terminal region of RIP2 contains four identified phosphorylation sites—S527, S529, S531 and S539.

**Table 2.** RIP2 phosphorylation events.

residue	reference
S168	Oppermann *et al.* [75]
S176	Dorsch *et al.* [76]
S363	Oppermann *et al.* [75] and Daub *et al.* [77]
Y381	Zhao *et al.* [78] and Tigno-Aranjuez *et al*. [79]
S393	Oppermann *et al.* [75]
Y474	Tigno-Aranjuez *et al.* [79]
Y520	Tigno-Aranjuez *et al.* [79]
S527	Oppermann *et al.* [75] and Daub *et al.* [77]
S529	Olsen *et al.* [80]
S531	Dephoure *et al.* [81]
S539	Oppermann *et al.* [75] and Daub *et al.* [77]

One phosphorylation event which is an exception to this trend occurs at Y474, which falls in helix 3 of the RIP2 CARD and corresponds to one of the key residues used by Apaf-1 to form a type I interaction with procaspase-9. Mutation of this tyrosine in RIP2 reduces its ability to bind either NOD1 or NOD2 [67,79]. It is conceivable that phosphorylation here may be used to provide a negative charge for engaging an opposing, basic surface. Intriguingly, the corresponding tyrosine residue is phosphorylated in apoptosis-associated speck-like protein containing a CARD (ASC), and this phosphorylation is described as a switch that controls ASC speck formation [82]. It follows that phosphorylation of RIP2 on Y474 could play a similar role in directing the assembly of a CARD complex downstream of NOD1 and NOD2. Various other CARDs have a tyrosine in this position, and phosphorylation may play a broad role in controlling type I CARD : CARD interactions.

At least five E3 ligases have been implicated recently in the ubiquitination of RIP2: ITCH [83], cIAP1 [84], cIAP2 [84], XIAP [85,86] and Pellino3 [87]. The ubiquitination events mediated by the cIAPs, XIAP and Pellino3 are reported to increase signalling to the NF-κB and JNK pathways, whereas ubiquitination by ITCH reduces signalling to NF-κB in favour of JNK and MAPK phosphorylation. A concurrent study of XIAP and Pellino3 concluded that the two E3 ligases act through different mechanisms, because XIAP acted by recruiting Sharpin and Pellino3 did not [87]. K209 has been identified as an important ubiquitination site on RIP2 [88], but the E3 ligase responsible for this ubiquitination has not been determined, though ITCH and Pellino3 have been ruled out [87]. The K209 residue is conserved in RIP1 and RIP4 and may play a similar role in these proteins.

## 7. Signal transduction downstream of RIP2

**Figure 3.**
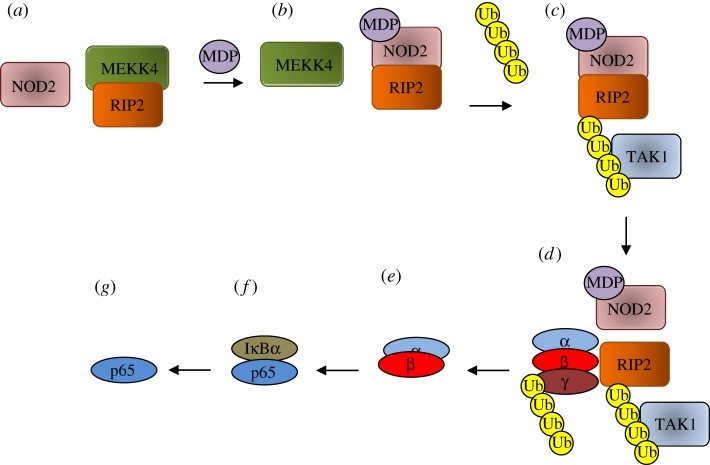
Signal transduction downstream of RIP2. (*a*) MEKK4 and RIP2 form a complex preventing RIP2 ubiquitination. (*b*) In the presence of MDP, the MEKK4 : RIP2 complex dissociates and a NOD2 : RIP2 complex is able to form. (*c*) RIP2 is then ubiquitinated, leading to the recruitment of TAK1. (*d*) The IKK complex is recruited to RIP2 and IKKγ is ubiquitinated by TAK1. (*e*) IKKγ is then degraded by the proteasome, relieving inhibition of IKKα/β. (*f*) IKKα/β phosphorylates IκBα which subsequently releases p65 (*g*), which then enters the nucleus to enhance transcription of inflammatory cytokines.

While NOD1 and NOD2 are autoinhibited until ligand activation, it has been suggested that RIP2 activity is kept in check through an interaction with MEKK4. In this sequestration model (figure 3) [89], a MEKK4 : RIP2 complex exists under basal conditions in the absence or presence of NOD2. When MDP is introduced into this system, the MEKK4 : RIP2 complex dissociates and a NOD2 : RIP2 complex forms. Until NOD2 is introduced, MEKK4 : RIP2 is stable in the presence of MDP, suggesting that activated NOD2 competes for RIP2. This basal inhibition of the NOD2 : RIP2 complex formation translates to an inhibition of NOD2–NF-κB signalling while still allowing the activation of JNK by RIP2.

Following the release of RIP2 from the MEKK4 complex and its binding to NOD2, it is ubiquitinated, leading to the recruitment of TAK1 to its kinase domain. Simultaneous binding of the IKK complex to the RIP2 intermediate domain results in ubiquitination of IKKγ (NEMO) by TAK1 and its degradation, which allows the IKKα and IKKβ subunits to phosphorylate IκBα. Phosphorylated IκBα is degraded, releasing p65 and allowing its transport into the nucleus, where it affects transcription (figure 3) [88,90].

## 8. NOD2 and autophagy

Evolving from a stress response in unicellular organisms, autophagy is a bulk degradation system which economizes resources under harsh conditions. While beyond the scope of this review, this process is covered extensively elsewhere [91,92]. In multicellular organisms, it is becoming evident that autophagy has developed into a system capable of eliciting anti-microbial properties and is beginning to be connected to the influence of multiple pattern recognition receptors. Interactions between pattern recognition receptors and the autophagy machinery, such as NOD1 and NOD2 with ATG16L1 [7], RIG-I with the ATG5–ATG12 conjugate [93] and the NLRs NLRC4, NLRP3, NLRP4, NLRP10 and NOD2 with Beclin-1 [14], establish a firm link between pathogen detection and pathogen elimination.

The role of NOD1 and NOD2 in autophagy is a recent discovery and to date is still in the early stages of investigation. Multiple groups have shown NOD1- or NOD2-dependent activation of autophagy when cells are stimulated with their respective ligands [7,94]. However, the precise role that NOD1 and NOD2 play in activating autophagy is currently an area of controversy that may well show cell-dependent phenotypic effects as outlined below.

An original study by Travassos *et al.* [7] suggested autophagy was activated independently of RIP2 and NF-κB signalling, instead showing an interaction between ATG16L1 and the NODs. ATG16L1 is an essential component of the autophagic machinery, forming a complex with ATG5–ATG12 conjugates which function to dictate the site of LC3 lipidation in pre-autophagic structures [95,96]. HeLa cells transfected with ATG16L1 and NODs were infected with *Shigella flexneri* and displayed colocalization of the proteins at bacterial entry sites. This membrane colocalization was abrogated when NOD2 was replaced by the inactive, cytosolic frameshift SNP, fs1007insC, which retained ATG16L1 in the cytosol. Therefore, this model suggests that ATG16L1 is recruited to bacterial entry sites by NOD2, pinpointing the localization of the autophagic machinery [7]. A link between NOD2 and ATG16L1 as presented in this model is of great fundamental importance, because SNPs in both proteins are implicated in Crohn's disease and hamper autophagy induction [5,7,12].

To further support this theory, an interaction between NOD1 or NOD2 with ATG16L1 has been shown using recombinant protein pulldowns, implicating the CARDs of the NODs and the WD40 repeats of ATG16L1 as the interacting domains [11]. A short stretch of peptides in NOD2 CARDa has been suggested to mediate this interaction. This is a binding motif which is also present in other ATG16L1 binding proteins such as TMEM59 and TLR2 [12]. However, this motif is absent from NOD1 and indeed no interaction between NOD1 and ATG16L1 was reported, leading to a discrepancy with the work of Travassos *et al*. [7]. Comparative structural analysis suggests that the crucial residues identified for this motif in NOD2 form part of the conserved hydrophobic core, and so further analysis is necessary to definitively show its involvement in binding.

More recently, increasing evidence has been gathered to suggest an alternative mechanism of autophagy activation which requires RIP2 [94,97–99]. Homer *et al.* [98] have demonstrated the crucial nature of RIP2 kinase activity in relation to autophagy. Treatment of HCT116 endothelial cells with erlotinib, a RIP2 tyrosine kinase inhibitor, impeded LC3-II accumulation on autophagic membranes. While all studies so far have demonstrated the dispensable nature of NF-κB signalling to autophagy [7,89,100], Homer *et al*. have shown that the MAPK p38 is required for bacterial clearance. A RIP2 binding partner, MEKK4, helps to dictate whether NOD2 signals through NF-κB or MAPK pathways [89]. Because MEKK4 is also essential for autophagy activation [98], this indicates that NOD2 activates autophagy through a RIP2 pathway dependent upon MEKK4 activation of p38. This pathway is negatively regulated by PP2A, which acts on an unknown target downstream of p38. Upon stimulation by MDP, PP2A itself becomes phosphorylated and downregulated in a process dependent upon RIP2 kinase activity [98]. Whereas this report indicated that other MAPKs such as JNK and ERK1 were not involved in an autophagic response, Anand *et al*. [97] observed a marked reduction in autophagic clearance of *Listeria monocytogenes* in dendritic cells defective in ERK1. The differences seen in autophagic response and MAPK involvement may in part be due to the different cell types used in these studies [7,94,97–99,101], but further work is needed for clarification.

## 9. The importance of membrane localization

NLRs are classically defined as cytosolic detectors in the immune response. However, NOD1 and NOD2 partially localize to the plasma and endosomal membrane of cells in which they are endogenously expressed, a characteristic which is replicated in cells where NODs have been overexpressed [45,100–102]. The exact mechanistic reason for this redistribution of NODs towards the membrane remains unclear but proposals include positioning of NODs to sites of ligand entry [45], activation of autophagy at bacterial entry sites [7] and regulation of NODs by binding partners positioned at the membrane [25,26].

Recruitment of NOD2 to the membrane is dependent upon Rac1, a Rho family GTPase. Rac1 and NOD2 co-immunoprecipitate and co-localize at membrane ruffles, but if Rac1 is knocked down then NOD2 membrane localization is abrogated [13,40,103,104]. Classically, Rac1's function has been defined as modulation of the actin cytoskeleton, leading to cell movement and membrane protrusions [105]. However, it has also been implicated in cell proliferation, cell adhesion, phagocytosis, interleukin production, superoxide production and transcriptional regulation [106]. *Salmonella* infection of intestinal epithelium cells is mediated by its manipulation of host cell machinery, including Rac1, reorganizing the actin cytoskeleton and allowing bacterial penetration of the cell [107]. Therefore, a link between NOD2 and Rac1 connects it to bacterial entry sites. It is unclear how Rac1 affects NOD2 membrane localization, but, because disruption of the actin cytoskeleton also reduces the amount of NOD2 at the membrane, it is possibly in an actin-dependent manner [13,40,103,104]. Interestingly, NOD2 has also been implicated in an interaction with CD147, a transmembrane glycoprotein found to increase the invasiveness of *L. monocytogenes* [21]*.* Again, because invasion of this bacterium into cells will require reorganization of the actin cytoskeleton, the link between NOD2 and CD147 therefore serves to reinforce the connection between bacterial invasion, the cytoskeleton and NOD2.

NOD2 binds to the peripheral membrane protein FERM and PDZ-domain-containing 2 (FRMPD2), anchoring itself to the membrane [26]. FRMPD2 is formed of an N-terminal KIND domain, a central membrane-binding FERM domain and three PDZ domains at the C-terminus functioning to steer the protein to the membrane [108]. NOD2 and FRMPD2 co-immunoprecipitate and co-localize together at the membrane, an interaction which is mediated by the LRR domain of NOD2 and the FERM and PDZ 2 domains of FRMPD2. Knockdown of FRMPD2 reduces NOD2 presence at the membrane and reduces NOD2-induced NF-κB activity [26]. Because both FRMPD2 and Rac1 have been implicated in membrane recruitment of NOD2, it is unclear why FRMPD2 should lead to an enhancement of activity whereas Rac1 has a negative impact [13,40].

Once at the membrane, NOD2 interacts with a myriad of additional binding partners. One possible reason for the negative impact of Rac1 on NOD2 signalling [13] is the juxtaposition of NOD2 with negative regulators such as Erbin. This is a transmembrane protein which both co-localizes with NOD2 at bacterial entry sites and co-immunoprecipitates with it [24,25]. The pulldown of Erbin with NOD2 is enhanced during infection; however, NOD2 is not dependent on Erbin for membrane localization. Erbin has a negative impact on NOD2-mediated NF-κB activity and may explain why disruption of NOD2 membrane recruitment by inhibiting Rac1 led to increased NOD signalling [24,25]. In addition to Erbin, AAMP, CD147 and RIG-I all reportedly bind to NOD2 and elicit a negative impact on NF-κB signalling.

Membrane-localized proteins can also have a positive effect on NOD1/2 signalling. As well as FRMPD2, DUOX2 is also reported to enhance NF-κB signalling [23,26]. In addition, the interaction of NOD2 and DUOX2 is reported to be responsible for the NOD2-dependent reactive oxygen species (ROS) production seen in Caco-2 cells following MDP treatment. As well as enhancing NF-κB activity, this ROS production was found to be important for protection from *L. monocytogenes* [23].

NOD2 has been implicated in recruiting binding partners to the membrane, forming active signalling complexes. A pool of RIP2 is recruited to the membrane in a NOD2-dependent fashion [43] and, similarly, ATG16L1 may also be recruited to bacterial entry sites to induce autophagy [7]. So, while membrane recruitment may be important for fine-tuning of NOD2 activity, it is also crucial for directing bacterial killing.

## 10. Conclusion

It is certain that proteins will continue to be identified that influence NOD2 signalling, either through direct interaction with the receptor, via a more general interaction with the signalling complex, or in more abstract manners. As this review has highlighted, while some proteins are unique in their influence upon the NOD2 signalling pathway, there are others which also influence NOD1 signalling. Without doubt, newly identified proteins will fall into both categories. Understanding what these cellular proteins are doing and indeed why they are doing it is of paramount importance if we are to be able to firstly understand NOD2 function, and second, specifically modulate the NOD2 signalling pathway for therapeutic purposes. This is particularly important if we are to differentiate between the modulation of NOD1 and NOD2 signalling. Indeed, we may find that it is through gaining an understanding of the role of proteins that influence either NOD2 or NOD1 signalling that we really begin to understand the different mechanisms of signalling activation and regulation between these two closely related proteins. If we ignore the role and function of these associated proteins in favour of focusing solely on the perceived key players of NOD2 and RIP2, we may miss opportunities for subtle, or specific, modulation and run the risk of inadvertently affecting a wide range of cellular processes. We also need to resolve the precise role of these proteins in relation to particular cell types, under certain conditions of stimulation, and in different species before we can be confident that we really understand the cellular and molecular basis of NOD2 signalling.
